# Bioresorbable Material in Secondary Orbital Reconstruction Surgery

**DOI:** 10.1155/2019/8715314

**Published:** 2019-02-03

**Authors:** Hui Pan, Zhenzhen Zhang, Weiwei Tang, Zhengkang Li, Yuan Deng

**Affiliations:** ^1^Department of Ophthalmology, Shanghai Ninth People's Hospital, Shanghai Key Laboratory of Orbital Diseases and Ocular Oncology, Shanghai Jiao Tong University School of Medicine, Shanghai, China; ^2^Department of Ophthalmology, The Second Affiliated Hospital of Anhui Medical University, Hefei, Anhui, China

## Abstract

**Purpose:**

To validate the potential of bioresorbable implantation in secondary revisional reconstruction after inadequate primary orbital fracture repair, with assessment of pre- and postoperative clinical characteristics and computed tomography image findings.

**Methods:**

A retrospective chart review was conducted on 16 consecutive patients treated for orbital fractures at Shanghai Ninth People's Hospital, Shanghai Jiao Tong University School of Medicine, with inadequate prior surgeries between July 2010 and June 2017; patients who had suffered orbital blowout fractures had undergone primary surgeries elsewhere. Secondary repair of orbital fractures used bioresorbable material following unsatisfactory primary orbital repair. Patients' demographics, degree of enophthalmos, ocular motility, diplopia test results, primary implants, and surgical complications were reviewed.

**Results:**

All 16 patients had primary orbital implants consisting of Medpor, titanium mesh, hydroxyapatite, or poly-L-lactide. Of the 16 cases, 14 had malpositioned implants posteriorly and two had implant infections. Findings following primary surgery included enophthalmos (12/16), diplopia (9/16), intraorbital abscess (2/16), and ocular movement pain (1/16). Mean preoperative enophthalmos was 3.8 ± 0.8 mm. Secondary reconstruction resulted in a mean reduction of enophthalmos by 3.1 ± 0.9 mm (*P* < 0.01). Nine in ten patients experienced improvements in postoperative ocular motility and diplopia following secondary surgery. Intraorbital abscesses and eyeball movement-associated pain were cured.

**Conclusions:**

This study demonstrates that secondary orbital reconstruction of previously repaired orbital fractures using bioresorbable material can achieve excellent functional and aesthetic results with minimal complications. Bioresorbable material should be considered in secondary orbital reconstruction when clinically indicated.

## 1. Introduction

Orbital blowout fractures are common and may occur in isolation or as part of a more complex facial fracture. Reconstruction requires orbital defect assessment and accurate restoration of orbital volume to prevent undesired outcomes of enophthalmos or diplopia [1]. Several publications on the subject of secondary orbital and periorbital fracture repairs did not involve prior surgical repair of medial or orbital floor walls [2, 3] or did not discuss the challenges and techniques of performing secondary revision surgery by removal of the implant or placement of new implants above a malpositioned implant [4, 5]. Inadequate primary orbital repair causing functional defects and cosmetic deficits is a great challenge for those surgeons who decide to perform secondary reconstruction surgery. Scarring accompanying the primary incision and fibrosis in response to implanted orbital materials increase the risks of complications associated with secondary surgery [6]. Nevertheless, if secondary surgery is performed, it can substantially improve patients' quality of life.

A previous study showed that porous polyethylene sheets with embedded titanium mesh (Medpor Titan) used in secondary orbital reconstruction in ten patients achieved functional and cosmetic improvement [2]. However, such implants are permanent foreign bodies and are hence susceptible to infection, hemorrhage, migration, and exposure over time [7, 8]. Bioresorbable implants have the advantages that they are (1) easy to contour (thermolabile implants); (2) provide mechanical integrity while the polymer resorbs; and (3) cause no donor-site morbidity [9]. Consequently, bioresorbable materials have been used for reconstruction of orbital defects [9–11]. However validation of these bioresorbable materials for use in secondary orbital reconstruction has not been reported in the literature.

In this retrospective study, bioresorbable poly-L/DL-lactide (85% : 15%) implants (RapidSorb) were used for secondary repair of the orbital medial and/or floor walls. The clinical characteristics, preoperative imaging findings, and pre- and postoperative surgical outcomes of patients who underwent secondary reconstruction were evaluated following unsatisfactory primary orbital fracture repair.

## 2. Methods

The institutional review committee of Shanghai Ninth People's Hospital, Shanghai Jiao Tong University School of Medicine, approved this retrospective review with waiver of informed consent. Patient consent to publish identifiable photograph archival statement was obtained from the all study participants. All patients who underwent secondary revision surgery in our center following primary orbital fracture repair elsewhere were reviewed. Sixteen patients treated between July 2010 and June 2017 with a minimum follow-up period of 12 months were included in the study. Medical records obtained included patient age, gender, mechanism of initial injury, orbital fracture location, implants used in previous surgery, complications after primary surgery or indication for secondary surgery, implant used in secondary repair, and interval between primary and secondary surgery.

Exophthalmometry was performed using a Hertel exophthalmometer. Preoperative and postoperative CT scans of the orbits were also obtained and evaluated for implant placement and position. Limitation of extraocular muscle movement (EOM) and diplopia was measured by a synoptophore examination. Eye motility was assessed in supraduction, infraduction, adduction, and abduction. Duction limitation was graded on a scale from 0 (no limitation), −1 (duction 30°–45°), −2 (15°–30°), and −3 (duction <15°) to −4 (no movement) [12, 13]. Enophthalmos was compared pre- and postoperatively using paired *t*-tests for statistical analysis with SPSS 20, where statistical significance was set at *P* < 0.05.

To evaluate implant malposition as part of preoperative planning, CT scans before secondary revision surgery were obtained with at least 0.625 mm slice thickness and used for preoperative planning and simulation. Sixteen cases of orbital medial or/and floor walls were reconstructed using a square plate of 85 : 15 poly(L-lactide-co-glycolide) (RapidSorb, Synthes, Oberdorf, Switzerland) by a transconjunctival approach. The plate was 50 mm in width and 0.8 mm in thickness. Following incubation in a bath of 65–75°C sterile saline, the implant was contoured onto the orbit of a three-dimensional (3D) skull model at bending temperatures, and then the plate was cut to the appropriate size according to its anatomical position using scissors. After explanting the previous implant and repositioning of orbital content, the desired shaped plate was fixed with bioresorbable screws (Synthes).

## 3. Results

Sixteen patients (nine males and seven females) underwent secondary orbital reconstruction after unsatisfactory primary repair. Mean age at the time of secondary surgery was 37 years, with a range of 14 to 67 years (Table 1). Orbital fractures occurred by various mechanisms and were the results of motor vehicle accidents (six cases), human assault (five cases), work-related accidents (three cases), and falls (two cases). Five cases were complex facial fractures involving the zygomaticomaxillary complex (ZMC) bone in addition to the orbital wall, ten cases were fractures involving the orbital medial wall and floor excluding the lateral or roof walls, and one case had an injury to the orbital floor wall alone (Table 1).

The majority of cases underwent primary surgical repair at a median of 1 month following trauma (range: 1 week to 4 months). All patients presented at our center seeking secondary revision of unsatisfactory previous surgical repair elsewhere at a median of 25 months after initial repair (range: 1 month to 8 years). Twelve of the 16 patients presented with enophthalmos, with a mean relative enophthalmos of 3.8 ± 0.8 mm. Nine patients had diplopia with restriction on duction. Two patients suffered intraorbital abscesses due to implant infection. Only one patient experienced eyeball movement pain. Other findings included infraorbital hypoesthesia or paresthesia (one case) and superior sulcus deformity (one case).

**Figure 1:**
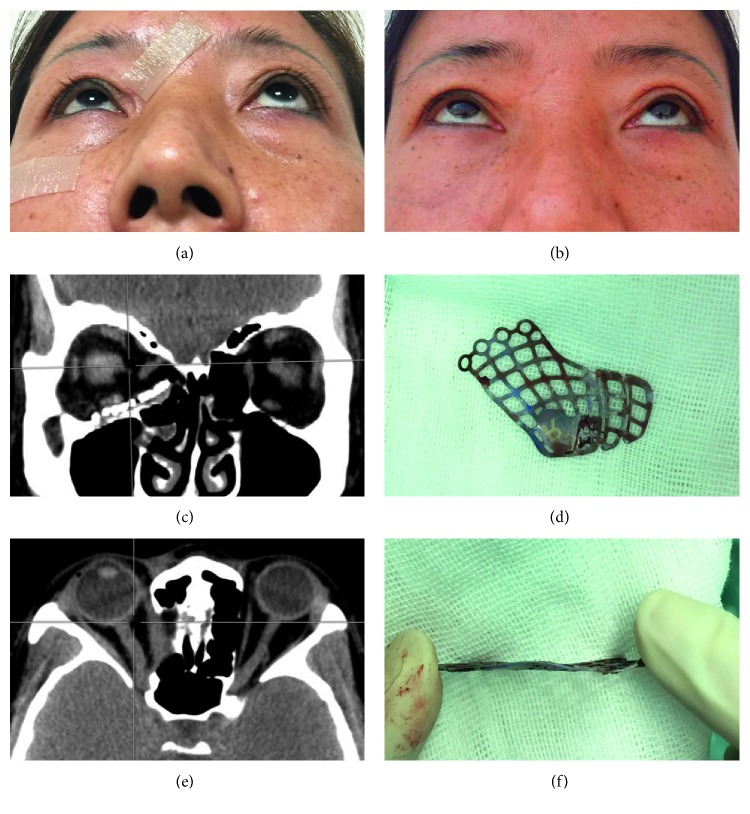
Case of a malpositioned titanium mesh implant. (a) Enophthalmos 1 month after previous orbital fracture repair. (b) The enophthalmos was fully corrected 6 months after secondary implantation of RapidSorb. (c and e) CT images showing that the implant did not fully cover the medial wall defect, causing herniation of orbital contents into the ethmoid sinus. (d and f) The explanted titanium mesh was flat and did not conform to the orbital anatomical structure.

**Figure 2:**
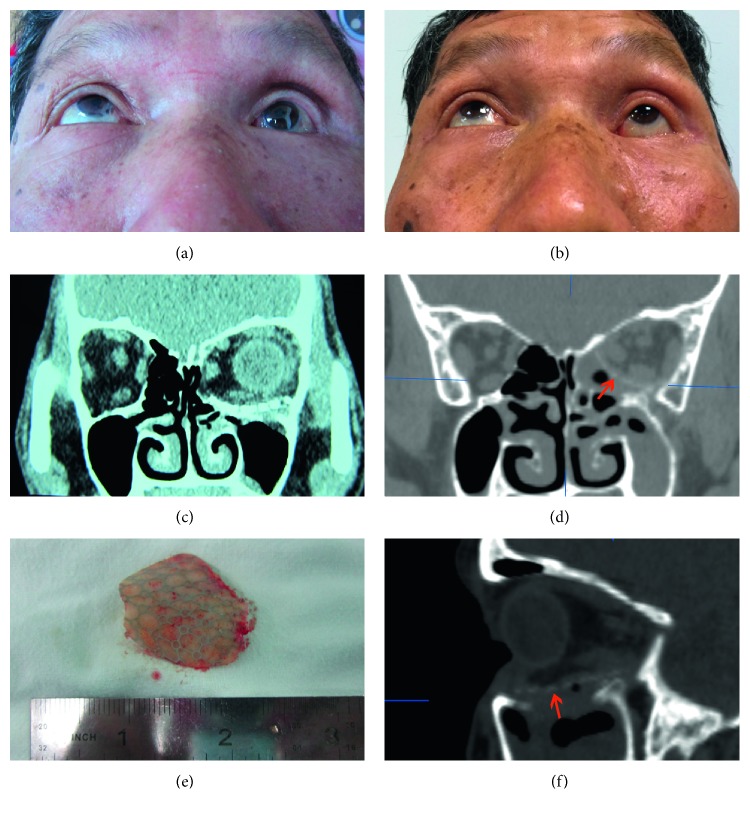
Case of a malpositioned Medpor Titan implant. (a) The left eye exhibited enophthalmos 5 months after primary surgery. (b) The enophthalmos was fully corrected over a 16 month follow-up. (c) The implant reconstructed only the wall of the orbital floor leaving the medial wall unrepaired. (e) The explanted Medpor Titan mesh. (d and f) A CT scan shows the newly positioned implant totally covering the orbital medial wall and floor defects (red arrow).

**Figure 3:**
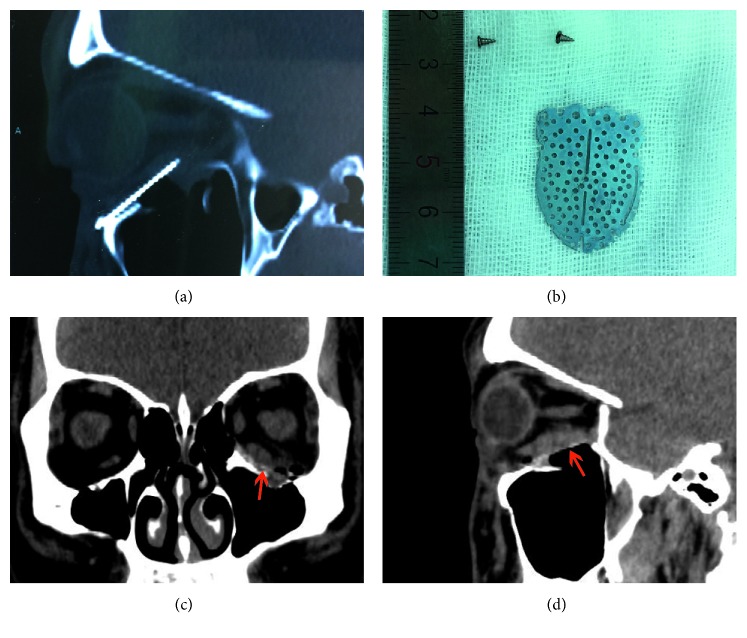
Case with severe ocular movement pain. (a) CT image showing the superiorly displaced floor implant and significantly incarcerated extraocular muscle. (b) The removed titanium mesh and screws. (c and d) Coronal and sagittal CT views of the implanted RapidSorb at 10 months postoperatively.

**Figure 4:**
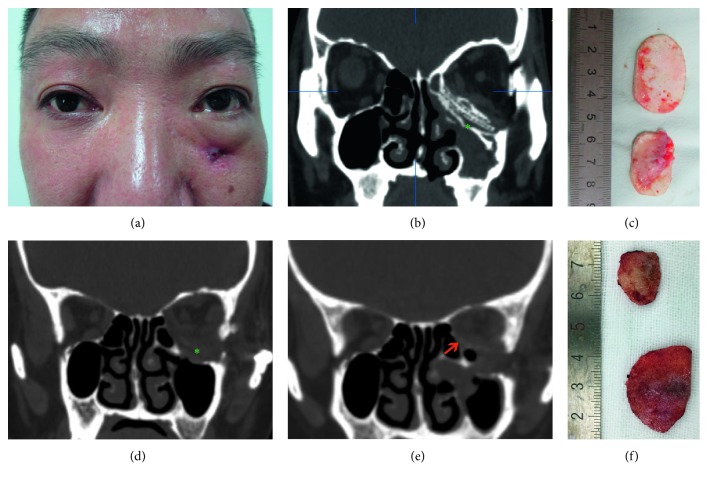
Two cases of implant infections. (a) Patient with infraorbital intermittent swelling and fistula 9 years after implantation of hydroxyapatite. (b and d) CT showing intraorbital abscesses in these two cases (asterisks). (c and f) The explanted implants. (e) Orbital walls were secondarily reconstructed using Rapidsorb in a one-stage procedure with removal of the previous implants (red arrow).

In all patients, implants were explanted and materials comprised porous polyethylene (Medpor; seven cases), titanium implants (five cases), porous polyethylene-coated titanium mesh (Medpor Titan; two cases), hydroxyapatite (one case), and poly-L-lactide (one case). New orbital implants of RapidSorb were placed to reconstruct the orbital wall defects after repositioning the prolapsed orbital contents (Table 1). Case one exhibited enophthalmos 1 month after prior orbital fracture repair (Figure 1(a)), with a CT image showing that the implant did not fully cover the medial wall defect, leading to herniation of the orbital contents into the ethmoid sinus (Figures 1(c) and 1(e)). The explanted titanium mesh was flat and did not conform to the orbital anatomical structure (Figures 1(d) and 1(f)). The enophthalmos was fully corrected after secondary implantation of RapidSorb (Figure 1(b)). Case 8 shows enophthalmos 5 months after primary surgery (Figure 2(a)), while a CT image shows that the implant only reconstructed the floor wall, leaving the medial wall unrepaired (Figure 2(c)). Following careful explanation of the Medpor Titan mesh (Figure 2(e)), the Rapidsorb was implanted. The CT scan shows the new implant totally covering the orbital medial wall and floor defects (Figures 2(d) and 2(f)), and the enophthalmos was fully corrected during the 16 month follow-up (Figure 2(b)). Case 7, with a superiorly displaced floor implant, caused incarceration of the extraocular muscle (Figure 3(a)) and significant pain with extraocular movements. After removal of the titanium mesh (Figure 3(b)) followed by implantation of the RapidSorb (Figures 3(c) and 3(d), red arrow), the patients were free from previous complications. Two cases developed intraorbital abscesses (Figures 4(b) and 4(d), asterisk) with intermittent infraorbital swelling and fistula 9 years (Figure 4(a)) after implantation of hydroxyapatite (Figure 4(c)) or 7 years after Medpor implantation (Figure 4(f)). The orbital walls were secondarily reconstructed using Rapidsorb in a one-stage operation combined with removal of previous implants (Figure 4(e), red arrow).

Mean follow-up was 18.4 ± 5.9 months. Secondary reconstruction resulted in a mean enophthalmos reduction of 3.1 ± 0.9 mm (*P* < 0.01). All nine patients with restricted ocular motility had diplopia in some position of the gaze preoperatively. Of these, eight had complete resolution of diplopia postoperatively in the extremes of the gaze after intensive eye movement for 3 months. One patient had persistent diplopia on the down gaze as before. The patient with a superior sulcus deformity showed resolution following secondary reconstruction. Intraorbital abscesses in two patients with primary placed Medpor and hydroxyapatite showed no recurrence beyond 15 months postoperatively after secondary surgery. None of the patients had new persistent infraorbital hypoesthesia following secondary surgery.

## 4. Discussion

In this retrospective study, enophthalmos, diplopia, and limitation of EOM were frequent complications of prior orbital repair surgery. Previous reports showed that 27.5% of the patients had residual enophthalmos and 20%–37% of patients had postoperative diplopia after surgery [14–16]. The decision for secondary repair was based on clinical presentation correlated with radiographic findings, such as a malpositioned or absent orbital implant [6]. Secondary orbital reconstruction for unsatisfactory primary orbital repair can improve functional deficits and aesthetic results [6]. All patients treated in our study showed significant improvement in ocular motility, diplopia, and enophthalmos postoperatively. We propose that since secondary surgery for orbital blowout fracture is generally considered a technically demanding procedure, the surgical procedure must be managed carefully by experienced surgeons to lower the high rates of these complications as before [6, 14].

Alloplastic implant materials such as titanium mesh, porous polyethylene (Medpor), and hydroxyapatite provide good tensile strength, but are susceptible to infection, hemorrhage, migration, and exposure over a long period [17–19]. The bioresorbable material 85 : 15 poly (L-lactide-co-glycolide) (RapidSorb) is expected to fully degrade by around 12 months and has the advantages of being easy to contour (thermolabile implants), providing mechanical integrity while the polymer resorbs and having no donor-site morbidity [11]. While bioresorbable implants are radiologically visible, especially on a soft tissue window, in the early postoperative scans, they appear either as isodense or hyperdense plates on CT images [11]. Consequently, bioresorbable materials have been gaining popularity for the reconstruction of orbital defects [9, 11, 20]. One major concern about secondary orbital surgery involving explanting primary malpositions or infected implants is the selection of reconstruction material.

This study reports bioresorbable material (RapidSorb) used for secondary revision surgery in 14 cases of malpositioned materials and 2 cases of infected implants with assessment of pre- and postoperative clinical characteristics and computed tomography image findings. The results show that bioresorbable implants substantially improve functional deficits and facial disfigurement with acceptable sequelae during long-term follow-up. However, limitations of this study include its retrospective nature and limited sample size.

## 5. Conclusions

The results of our study indicated revisional surgery can restore the contours of secondary deformities caused by inadequate prior orbital fracture repairs when clinically indicated, and secondary orbital reconstruction using bioresorbable materials can provide excellent functional and cosmetic results with minimal complications.

## Figures and Tables

**Table 1:** Patient demographics.

Case	Fracture location	Primary interval	Primary implants	Primary implant site	Indication for secondary surgery	Secondary interval	Secondary implant site
1	M + F	4 months	Titanium	F	Enophthalmos, diplopia	1 month	M + F
2	M + F, ZMC	1 month	Titanium	F, ZMC	Enophthalmos	4 months	M + F
3	M + F, ZMC	2 months	Medpor	F	Intraorbital abscess	7 years	M + F
4	M + F	1 month	Medpor	F	Enophthalmos diplopia	1 month	M + F
5	M + F	1 month	Hydroxyapatite	M + F	Intraorbital abscess	8 years	M + F
6	M + F	1 month	Medpor	M + F	Enophthalmos	5 years	M + F
7	M + F	1 month	Titanium	F	Ocular movement pain, diplopia	2 weeks	M + F
8	M + F	1 week	Medpor Titan	M + F	Enophthalmos, diplopia	5 months	M + F
9	M + F	1 week	Medpor Titan	M + F	Enophthalmos, diplopia	4 years	M + F
10	F, ZMC	10 days	Titanium	F, ZMC	Enophthalmos	1 year	F
11	M + F	1 week	Medpor	F	Enophthalmos, diplopia	1 year	M + F
12	M + F	1 week	Medpor	F	Diplopia	3 months	M + F
13	M + F	25 days	Medpor	M + F	Enophthalmos, diplopia	6 months	M + F
14	F	1 month	Titanium	F	Enophthalmos	1 month	F
15	M + F, ZMC	1 month	Poly-L-lactide	F, ZMC	Enophthalmos, diplopia	4 months	M + F
16	M + F, ZMC	1 month	Medpor	F	Enophthalmos	5 years	M + F

Primary interval: time between injury and primary repair; secondary interval: time between primary and secondary surgery; Medpor Titan mesh: porous polyethylene sheets with embedded titanium mesh; M: medial wall; F: orbital floor; ZMC: zygomaticomaxillary complex.

## Data Availability

The CT data used to support the findings of this study are available from the corresponding author upon request.
